# *Cordyceps* Polysaccharides: A Review of Their Immunomodulatory Effects

**DOI:** 10.3390/molecules29215107

**Published:** 2024-10-29

**Authors:** Liping Chen, Xiao Liu, Kaiyue Zheng, Yang Wang, Minglong Li, Yuyu Zhang, Yuan Cui, Sichun Deng, Shiqi Liu, Gaoju Zhang, Ling Li, Yuxin He

**Affiliations:** 1 School of Comprehensive Health Management, Xihua University, Chengdu 610097, China; lpingchen2022@163.com; 2 School of Food and Bioengineering, Xihua University, Chengdu 610097, China; 13086331382@163.com (X.L.); 17767916603@163.com (K.Z.); wy15008313734@163.com (Y.W.); cyylml123456@126.com (M.L.); zhangyuyu7626@163.com (Y.Z.); cy@stu.xhu.edu.cn (Y.C.); 19150311972@163.com (S.D.); 13699066740@163.com (S.L.); 3 Sichuan Chinese Herb Preparation, Chengdu 611732, China; zgj0175@163.com

**Keywords:** *Cordyceps*, *Cordyceps sinensis*, *Cordyceps militaris*, polysaccharide, immune modulation

## Abstract

*Cordyceps* primarily consists of ascomycetes, a parasitic fungus that infects insects and arthropods. Recently, *Cordyceps* has been shown to manifest a diverse range of pharmacological activities, rendering it applicable for the treatment and mitigation of various diseases, such as diabetes, acute liver injury, and colitis. Many active constituents have been identified from *Cordyceps sinensis*, including cordycepin, adenosine, sterols, and polysaccharides. Polysaccharides constitute a primary active component of *Cordyceps*, exhibiting immunomodulatory effects. We searched the Web of Science database with the keywords of cordyceps, polysaccharide, and immune modulation; collected related studies from 2004 to 2024; and eliminated articles with low influence and workload. A review of the research advancements regarding the immunomodulatory effects of *Cordyceps* polysaccharides was conducted with the aim of furnishing valuable reference information. Research indicates that polysaccharides exhibiting immunomodulatory activity are predominantly sourced from *Cordyceps sinensis* and *Cordyceps militaris*. Immunological experimental results demonstrate that *Cordyceps* polysaccharides can augment the activities of macrophages, lymphocytes, and dendritic cells while fostering the expression of immune-active substances such as cytokines and chemokines. Furthermore, animal experiments have substantiated the immunomodulatory effects of *Cordyceps* polysaccharides. These effects encompass ameliorating immune suppression induced by drugs or radiation, enhancing immune organ indices, elevating the expression of immunoreactive substances, and mitigating immune evasion prompted by tumors. In conclusion, *Cordyceps* polysaccharides exhibit significant immunomodulatory activity and merit further investigation.

## 1. Introduction

*Cordyceps* predominantly comprises ascomycetes parasitic fungi, which flourish through parasitism in insects and arthropods, encompassing orders such as *Coleoptera*, *Hemiptera*, *Hymenoptera*, *Lepidoptera*, and *Diptera*. Furthermore, a minor fraction of *Cordyceps* exhibits parasitic behavior toward other fungi [1]. The diversity in parasitic fungi and their corresponding hosts contributes to the varied forms observed within the genus *Cordyceps*. Consequently, the *Cordyceps* genus presently comprises over 750 identified species [2]. In the genus *Cordyceps*, *Cordyceps sinensis* (the new species name is *Ophiocordyceps sinensis*) and *Cordyceps militaris* emerge prominently as the two most renowned and widely utilized species [3]. Empirical evidence indicates that these species have demonstrated utility in the treatment and amelioration of a spectrum of diseases, encompassing, but not limited to, cancer, diabetes, cardiovascular disease, liver damage, and kidney damage [4,5]. This has garnered global interest in utilizing *Cordyceps* and their derivatives for medicinal, dietary, or tonic purposes. 

With the increasing attention directed toward *Cordyceps*, extensive research has been undertaken to investigate its bioactive components. Polysaccharides are acknowledged as principal bioactive constituents in *Cordyceps* [6]. Mounting evidence from pharmacological studies indicates that *Cordyceps* polysaccharides exhibit a diverse array of biological activities [7]. Among these, the immunomodulatory activity of *Cordyceps* polysaccharides stands out prominently [8]. Hence, with the aim of furnishing scholars with comprehensive and valuable insights, this article reviews the immunomodulatory effects of *Cordyceps* polysaccharides, encompassing both the polysaccharide fraction and purified polysaccharides characterized by a singular structure. These polysaccharides comprise intracellular polysaccharides extracted from fruiting bodies or mycelium, as well as extracellular polysaccharides extracted from the fermentation broth of artificially cultivated *Cordyceps*. *Cordyceps sinensis* and *Cordyceps militaris* stand out as the two most extensively studied types of *Cordyceps* [9]. Moreover, several studies have indicated that polysaccharides extracted from *Cordyceps cicadae*, *Cordyceps kyushuensis*, *Cordyceps gunnii*, *Cordyceps taii*, and *Cordyceps sobolifera* also exhibit immunomodulatory activity [10]. Experimental findings reveal that both the polysaccharide fraction and purified polysaccharides exert immunomodulatory effects on various pivotal immune cells, such as macrophages, lymphocytes, and dendritic cells [11]. These effects encompass the augmentation of cellular activity, stimulation of cell proliferation, and enhancement of the expression of immune substances [12,13]. Furthermore, animal experiments demonstrate a significant enhancement in immune organ indices upon the administration of these polysaccharides [14,15]. *Cordyceps* polysaccharides exhibit efficacy in ameliorating immune suppression induced by drugs, radiation, and other factors, as well as in stimulating the immune system to combat tumors [16,17]. Presently, there is a lack of comprehensive discussion regarding the immunomodulatory effects of *Cordyceps* polysaccharides. In this study, we conducted a comprehensive analysis of the immunomodulatory effects of *Cordyceps* polysaccharides, providing a significant reference for elucidating their immunomodulatory mechanism, which deserves further exploration. 

## 2. Immunomodulatory Activity of Polysaccharides from *Cordyceps sinensis*

Owing to its extensive utilization and elevated market value, *Cordyceps sinensis* is the most extensively investigated species within the *Cordyceps* genus. In China, *Cordyceps sinensis* is even recognized as the sole species within the genus officially listed as a medicinal substance in the *Chinese Pharmacopoeia* [18]. As key bioactive constituents of *Cordyceps sinensis*, polysaccharides have garnered growing interest due to their immunomodulatory properties [9]. Presently, the predominant technique for extracting polysaccharides from *Cordyceps sinensis* involves the hot water extraction method. Methods such as ultrasonic-assisted and microwave-assisted extraction are also employed to improve extraction efficiency [19]. The principal purification techniques for *Cordyceps sinensis* polysaccharides encompass ion exchange chromatography and gel chromatography [20]. Chemical structure characterization involves techniques such as gas chromatography–mass spectrometry, high-performance liquid chromatography, infrared spectroscopy, and nuclear magnetic resonance, which are employed for analyzing structural features of polysaccharides, including molecular weight, configuration of glycosidic bonds, and monosaccharide composition [21]. To date, besides the polysaccharide fraction, 17 purified polysaccharides characterized by a singular structure have been confirmed to exhibit immunomodulatory activity. Their chemical structures are summarized in Table 1.

### 2.1. Immunomodulatory Effects of Intracellular Polysaccharides on Immune Cells

Macrophages play a pivotal role in the immune process, possessing the capability to phagocytose and digest diverse pathogens. Furthermore, macrophages exhibit the capacity to activate other immune cells, particularly T lymphocytes, through processes such as antigen presentation and the release of cytokines, thereby instigating specific immune responses. In essence, the multifaceted functions of macrophages render them an indispensable and integral component of the immune system [57]. Evidence suggested that the *Cordyceps sinensis* polysaccharide fraction (25–100 μg/mL) induced a shift in the M2 phenotype of Ana-1 macrophages to the M1 phenotype. This shift activated macrophage immune activity, upregulating tumor necrosis factor-α (TNF-α), interleukin-12 (IL-12), and inducible nitric oxide synthase (iNOS) expression, while downregulating IL-10. Further research revealed that these effects were attributed to nuclear factor-κB (NF-κB) pathway activation and reduced expression of the mannose receptor and scavenger receptor [58]. Chen et al. and Meng et al. reported that the *Cordyceps sinensis* polysaccharide fraction (30–300 μg/mL) enhanced the phagocytic activity of RAW264.7 macrophages. Additionally, it stimulated the release of NO and cytokines IL-1α, IL-1β, IL-10, and TNF-α through the activation of the IκB/NF-κB pathway [59,60]. The *Cordyceps sinensis* polysaccharide fraction (0.1–15 μg/mL) not only enhanced macrophage activity but also induced RAW 264.7 macrophage differentiation into dendritic cells. Moreover, it promoted the maturation of mouse JAWS II dendritic cells by increasing the surface levels of cluster of differentiation 80 (CD80), CD86, and major histocompatibility complex class II (MHC II) antigen presentation molecules [61]. These studies demonstrate that the polysaccharide fraction of *Cordyceps sinensis* activates macrophage immune activity, promotes the expression of immune substances, and induces differentiation, with the NF-κB pathway activation playing a key role in this process.

In addition to the polysaccharide fraction, some purified polysaccharides characterized by a singular structure have been discovered to exert activation effects on macrophages. Furthermore, characterization of the chain conformation of pure polysaccharides indicates that they are predominantly composed of glucans. The polysaccharide NCSP-50 is a glucan with a molecular weight of 976 kDa. It comprises a main chain of (1→4)-linked α-D-Glc*p* with a single α-D-Glc*p* branch substituted at C-6. The immunomodulatory experiments unveiled that NCSP-50 (25–200 μg/mL) induced the proliferation of RAW 264.7 cells and augmented the production of NO, along with the cytokines IL-1β and TNF-α [34]. The polysaccharide CCP is a glucan with a molecular weight of 433.788 kDa, composed of non-reducing terminal D-Glc*p*, (1→4)-linked D-Glc*p*, and (1→4,6)-linked D-Glc*p* residues. Both Li et al. and Tan et al. have documented that CCP (1–400 μg/mL) induced the production of NO, IL-6, and TNF-α in RAW 264.7 cells through the activation of the toll-like receptor 4 (TLR4)/myeloid differentiation primary response 88 (MyD88)/p38 mitogen-activated protein kinase (p38) signaling pathway [22,62]. The polysaccharide HSWP-2a is a glucan with a molecular weight of 870.70 kDa. Further characterization of the chain conformation of HSWP-2 revealed that it is an α-(1→4)-D-glucan that branches at *O*-6, *O*-3, or *O*-2 with a terminal 1-linked α-D-Glc*p* as a side chain. HSWP-2a (25–200 μg/mL) augmented the phagocytic activity of RAW264.7 cells and elevated the production of NO, IL-1β, IL-6, and TNF-α through the activation of the p38, c-Jun N-terminal kinase (JNK), and NF-κB signaling pathways. Furthermore, HSWP-2a enhanced the proliferation of mouse splenic lymphocytes [32]. The polysaccharide UM01-S4 is a heteropolysaccharide with a molecular weight of 22.569 kDa. The monosaccharide composition includes mannose, glucose, galactose, and galacturonic acid with a molar ratio of 9.6:4.0:4.4:1.0. Characterization of the chain conformation of UM01-S4 indicated that it features a backbone of α-(1→2)-Man*p* with side chains consisting of β-(1→4)-Glc*p*, β-(1→2)-Gal*f*, terminal α-Gal*p*A, and α-Man*p*. Immunological findings suggested that UM01-S4 (0.1–3 μg/mL) boosted the proliferation and phagocytic activity of RAW 264.7 cells. Additionally, it stimulated the release of NO and cytokines IL-1β, IL-6, IL-12, and TNF-α through the activation of the mitogen-activated protein kinase (MAPK) and NF-κB signaling pathways [27]. The polysaccharide cordysinan is a hyperbranched heteropolysaccharide with a molecular weight of 22.37 kDa. Its monosaccharide composition comprises mannose, galactose, and glucose with a molar ratio of 4.4:3.8:1.0, with the primary backbone composed of 1,2-Man*p* residues. Cordysinan (10–100 μg/mL) induced the release of various cytokines, including IL-1β, IL-6, IL-10, and TNF-α, as well as chemokines such as monocyte chemoattractant protein-1 (MCP-1), macrophage inflammatory protein-1α (MIP-1α), interferon-inducible protein-10 (IP-10), and keratinocyte-derived chemokine (KC) from RAW 264.7 cells [26]. In summary, these studies collectively suggest that *Cordyceps sinensis* polysaccharides have the potential to enhance macrophage phagocytosis and stimulate cytokine and chemokine production by activating the MAPK and NF-κB signaling pathways. 

Hao et al. identified that in addition to exerting immunomodulatory effects on macrophages, the *Cordyceps sinensis* polysaccharide fraction counteracted immunosuppression in mouse spleen lymphocytes induced by simulated microgravity, promoted lymphocyte proliferation, and enhanced CD4 and CD8 expression. However, it was noteworthy that the promoting effect was observed at concentrations of 25 and 50 μg/mL, while above a concentration of 50 μg/mL, it transitioned to an inhibitory effect [63].

### 2.2. Immunomodulatory Effects of Intracellular Polysaccharides in Animal Models

Cyclophosphamide is a frequently employed chemotherapy drug known for its immunosuppressive effects. Its actions encompass inhibiting the activity of lymphocytes, attenuating the immune system’s memory response to immune stimuli, and diminishing cytokine production. Furthermore, cyclophosphamide can induce apoptosis in immune cells, leading to a reduction in their numbers [64]. Numerous scholars have explored the immunomodulatory effects of *Cordyceps sinensis* polysaccharides on cyclophosphamide-induced intestinal immune dysfunction. Fan et al. found that the *Cordyceps sinensis* polysaccharide fraction (200 mg/kg) increased histone H3 acetylation, which in turn mediated the specific expression of forkhead box protein P3 (Foxp3) in regulatory T cells, as well as decreased IL-17 and IL-21 expression in the colons of mice induced with cyclophosphamide [65]. Chen et al. discovered that the *Cordyceps sinensis* polysaccharide fraction (25–100 mg/kg) could enhance intestinal mucosal immunity and regulate the polarization of T helper type 1 (Th1)/Th2 cells in immunosuppressed cyclophosphamide-induced mice. The expression of Th1-style cytokines IL-2, IL-12 p40, interferon-γ (IFN-γ), and TNF-α, as well as Th2 cytokines IL-4, IL-10, and transcription factor GATA binding protein 3 (GATA-3) increased. Moreover, there was a rise in the formation of immunoglobulin A (IgA)-secreting cells and the content of secretory IgA [66]. Ying et al. also discovered that the *Cordyceps sinensis* polysaccharide fraction (25–100 mg/kg) could ameliorate cyclophosphamide-induced intestinal mucosal immunosuppression in mice. It stimulated the secretion of cytokines IL-12, IFN-γ, IL-4, IL-13, IL-6, IL-17, IL-10, transforming growth factor-β3 (TGF-β3), TNF-α, IL-2, and IL-21, as well as the production of transcription factors T-box expressed in T cells (T-bet), GATA-3, retinoic acid receptor-related orphan receptor γT (RORγt), and Foxp3 through the activation of the TLR/NF-κB signaling pathway [67]. In addition to the polysaccharide fraction, glucan NCSP-50 (25–100 mg/kg) could ameliorate cyclophosphamide-induced intestinal damage in mice by elevating the number of CD4+ T cells, modulating the expression of TLRs, and increasing the levels of cytokines associated with Th17 cells (IL-17 and IL-21) and Treg cells (TGF-β3) in the small intestine [68]. Taken together, these studies indicate that *Cordyceps sinensis* polysaccharides improve cyclophosphamide-induced intestinal immunosuppression by activating T-cell immune activity and inducing the expression of multiple cytokines and chemokines.

In addition to ameliorating cyclophosphamide-induced immunosuppression, *Cordyceps sinensis* polysaccharides have demonstrated the ability to inhibit radiation-induced immunosuppression. The *Cordyceps sinensis* polysaccharide fraction (50–200 mg/kg) enhanced the immune function of mice exposed to ^60^Co by improving lymphocyte proliferation and macrophage phagocytic activity. Additionally, it reduced the expression of IL-4 and IL-17 while increasing the expression of IL-5 [28]. Yang et al. also discovered that the *Cordyceps sinensis* polysaccharide fraction (100–400 mg/kg) could enhance survival rates and times in X-ray-irradiation-injured mice. It accelerated the recovery of white blood cells, increased the organ indices of the thymus and spleen, and elevated the DNA content in bone marrow cells by activating the MAPK signaling pathway [69]. In essence, *Cordyceps sinensis* polysaccharides ameliorate immune damage induced by radiation by reinstating both the quantity and functionality of immune cells.

Current research also indicates that *Cordyceps sinensis* polysaccharides can exert anti-cancer effects by enhancing immune function. Tan et al. discovered that the *Cordyceps sinensis* polysaccharide fraction (100–400 mg/kg) could elevate the thymus index, spleen index, and the numbers of CD4+, CD8+ T lymphocytes, and macrophages in the spleen. This inhibited the proliferation of cancer cells in H22 tumor-bearing mice [22].

Additionally, research indicates that *Cordyceps sinensis* polysaccharides have potential as a vaccine adjuvant. The polysaccharide PS is a heteropolysaccharide with a molecular weight of 83 kDa. The monosaccharide composition includes glucose, mannose, arabinose and galactose in a molar ratio of 8:90:1:1. Immunological experimental results demonstrated that PS (100–400 μ/mouse) could enhance the serum levels of IgG, IgG1, and IgG2b in ovalbumin (OVA)-immunized mice [35]. 

### 2.3. Immunomodulatory Effects of Extracellular Polysaccharides on Immune Cells

Due to the exorbitant cost of natural *Cordyceps sinensis*, scholars have endeavored to artificially cultivate *Cordyceps sinensis* to extract relevant biologically active substances. Strains isolated from natural *Cordyceps sinensis* are cultivated on a culture medium to obtain mycelium. The culture medium becomes enriched with extracellular polysaccharides produced by these *Cordyceps sinensis* fungi [70]. Current research indicates that, unlike intracellular polysaccharides primarily regulating macrophage activity, exopolysaccharides exhibit immunomodulatory effects on a diverse range of immune cells. These include macrophages, dendritic cells, and lymphocytes. 

Through isolation and purification, scholars have obtained three pure polysaccharides, AEPS-1, OSP, and HS002-II, all demonstrating immunomodulatory effects on macrophages. Immunological experimental results demonstrated that AEPS-1 (25–250 μg/mL), OSP (6.25–50 μg/mL), and HS002-II (0.2785–4.4 μM) enhanced the phagocytic activity of RAW264.7 cells and induced the production of NO and cytokines TNF-α, IL-6, and IL-1β. This was achieved by activating the MAPK, NF-κB, and phosphoinositide 3-kinase (PI3K)/protein kinase B (Akt) signaling pathways [44,45,46]. Chemical structure characterization revealed that AEPS-1 is an acidic glucan with a molecular weight of 36 kDa. It possesses a linear backbone of (1→3)-linked α-D-Glc*p* residues with two branches, α-D-Glc*p* and α-D-Glc*p*, attached to the main chain by (1→6) glycosidic bonds at every seventh α-D-Glc*p* unit [29]. The polysaccharide OSP is a heteropolysaccharide with a molecular weight of 27.972 kDa. Its monosaccharide composition includes xylose, mannose, glucose, and galactose in a ratio of 2.9:6.6:166:2.6 [25]. The molecular weight of the polysaccharide HS002-II is 44 kDa, and its monosaccharide composition is highly complex. It includes mannose, ribose, rhamnose, glucuronic acid, galacturonic acid, glucose, galactose, xylose, and arabinose in a molar ratio of 6.47:2.27:1.25:0.69:0.42:65.89:16.17:2.13:4.26. Characterization of the chain conformation of HS002-II revealed that it mainly comprises a long backbone of (1→3)-linked α-d-Rib*f* units, (1→4)-linked α-d-Xyl*p* units, and (1→4)-linked β-d-Glc*p* units, which was substituted at C-6 [33]. 

Dendritic cells are a crucial subset of immune cells with the primary function of presenting antigens and activating T lymphocytes [71]. Current research shows immune-activating effects on dendritic cells of two pure exopolysaccharides, namely, EPS and UST 2000. Chemical structure characterization revealed that the molecular weight of EPS is 104 kDa, and its monosaccharide composition comprises glucose and galactose in a ratio of 23:1:2.6. Immunological experimental results demonstrated that EPS (25–100 μg/mL) promoted the maturation and activation of mouse dendritic cells and increased the expression of IL-12, TNF-α, and iNOS and upregulated their capacity for antigen uptake and activation of CTLL-2 T lymphocyte proliferation. Subsequent investigations revealed that this was attributed to inhibition of signal transducer and activator of transcription 3 (STAT3) phosphorylation, leading to an increase in the expression of surface molecules such as MHC-II, CD40, CD80, and CD86 [72,73]. Sheng et al.’s study also verified that the activation of dendritic cells by EPS (25–100 μg/mL) heightened the proliferation of lymphocytes in the mouse spleen and thymus and elevated the expression of TNF-α, IFN-γ, and IL-2 [30]. In addition to EPS, the polysaccharide UST 2000 (6.25–100 μg/mL) promoted the proliferation of human T lymphocytes and the secretion of IL-2, IL-6, and IL-8 by activating the extracellular signal-regulated kinase (ERK) signaling pathway. Chemical structure characterization revealed that the molecular weight of UST 2000 is 82 kDa, and its monosaccharide composition comprises glucose, mannose, and galactose in a ratio of 2.4:2:1 [36]. In summary, these studies indicate that *Cordyceps sinensis* exopolysaccharides stimulate dendritic cell immune activity and induce T lymphocyte immune activation.

### 2.4. Immunomodulatory Effects of Extracellular Polysaccharides in Animal Models

The results of mouse immunosuppression experiments show that two pure extracellular polysaccharides, PHP and EPS, improve immune suppression by stimulating T lymphocyte immune activity and cytokine expression. The polysaccharide PHP is a water-soluble polysaccharide with a molecular weight of 58.14 kDa, and its monosaccharide composition includes mannose (2.49%), glucose (57.1%), galactose (1.43%), and galacturonic acid (0.321%). Immunological experimental results demonstrated that PHP (400 mg/kg) could restore the intestinal immune function of dextran sulfate sodium-induced mice by modulating the balance between Th17 and Treg cells. Specifically, the number of Th17 cells was reduced, while the number of Treg cells was increased [23]. The polysaccharide EPS is predominantly composed of glucose, accounting for approximately 99% of its structure. Characterization of the chain conformation of EPS revealed that it has a linear backbone of (1→3)-β-D-Glc*p* residues, featuring a single (1→6)-β-D-Glc*p* side-branching unit for every three β-D-Glc*p* residues. Immunological experimental results demonstrated that EPS (20 mg/kg) could increase the thymus and spleen indices and stimulate the release of TNF-α and INF-γ in cyclophosphamide-induced immunosuppressed mice [31].

## 3. Immunomodulatory Activity of Polysaccharides from *Cordyceps militaris*

*Cordyceps militaris* exhibits similar chemical capacities and medicinal functions to *Cordyceps sinensis,* but is more cost-effective, positioning it as the most viable substitute for *Cordyceps sinensis*. Therefore, within the *Cordyceps* genus, *Cordyceps militaris* enjoys a high degree of commercialization and research interest, second only to *Cordyceps sinensis* [74]. As primary active ingredients of *Cordyceps militaris*, polysaccharides have naturally garnered increasing attention due to their immunomodulatory activity. The processes of extraction, purification, and structural characterization of *Cordyceps militaris* polysaccharides closely resemble those of *Cordyceps sinensis* polysaccharides. Extraction methods encompass hot water extraction, subcritical water extraction, ultra-high-pressure extraction, microwave extraction, and ultrasonic extraction [75]. Purification methods involve anion exchange chromatography, gel filtration chromatography, and affinity chromatography. Chemical structure characterization encompasses methylation analysis, infrared spectroscopy, gas chromatography–mass spectrometry, and high-performance liquid chromatography [76]. To date, in addition to the polysaccharide fraction, 16 pure polysaccharides with immunomodulatory activity have been purified from *Cordyceps militaris*. Their chemical structures are summarized in Table 1.

### 3.1. Immunomodulatory Effects of Intracellular Polysaccharides on Immune Cells

Like *Cordyceps sinensis* intracellular polysaccharides, four pure intracellular polysaccharides isolated from *Cordyceps militaris* were demonstrated to exhibit immunomodulatory effects on macrophages, namely, CP2-S, CMP-Fr-II, CMP-III, and SDQCP-1. The polysaccharide CP2-S has a molecular weight of 1.328 kDa and is mainly composed of glucose. It (50–500 μg/mL) has been observed to stimulate phagocytosis, respiratory burst activity, and the secretion of NO, IL-1β, and IL-2 in RAW264.7 cells [24]. CMP-Fr-II is a water-soluble polysaccharide with a molecular weight of 126 kDa. The monosaccharide composition includes glucose, galactose, and mannose with a molar ratio of 56.4:26.4:17.2. It consists of (1→4) or (1→2)-linked Glc*p* or Gal*p* residues with a (1→2) or (1→6)-linked Man*p*, Glc*p*, or Gal*p* residue as a side chain. Immunological experimental results demonstrated that CMP-Fr-II (1000 µg/mL) upregulated the phenotypic functions of RAW264.7 cells and increased NO, TNF-α, and IL-1β production [42]. The molecular weight of the polysaccharide CMP-III is 49.76 kDa. Its monosaccharide composition includes glucose, mannose, and galactose with a molar ratio of 8.09:1.00:0.25. The main linkage types consist of (1→4)-α-D-Glc, (1→4,6)-α-D-Man, (1→)-α-D-Man, and (1→2,6)-α-D-Gal. Immunological experimental results demonstrated that CMP-III (50–500 μg/mL) enhanced phagocytosis and induced the production of NO, TNF-α, and IL-6 in RAW264.7 cells by activating the MAPK and NF-κB signaling pathways [45]. The molecular weight of the polysaccharide SDQCP-1 is 49.76 kDa. The monosaccharide composition includes mannose, glucose, and galactose in a molar ratio of 13.3:1.0:9.7. The backbone is composed of (1→2)-α-D-Man*p* and (1→4)-β-D-Glc*p* residues. Its side chains branch at the *O*-6 position of (1→2)-α-D-Man*p*, mainly via (1→2)-β-D-Gal*f* or (1→6)-α-D-Man*p* residues, which are terminated mainly with α-D-Gal*f* and α-D-Gal*p* residues. Immunological experimental results demonstrated that SDQCP-1 (50−250 µg/mL) induced M1 polarization of RAW 264.7 cells and stimulated the release of NO, TNF-α, IL-6, and IL-10 [46]. In summary, *Cordyceps militaris* polysaccharides exhibit regulatory effects on macrophages that are akin to those of *Cordyceps sinensis* polysaccharides, encompassing the activation of the MAPK and NF-κB signaling pathways, augmentation of immune factor release, and enhancement of phagocytic activity.

The THP-1 cell line is a human monocytic cell line that was originally isolated and established from the peripheral blood of patients with acute monocytic leukemia. It can be induced to differentiate into macrophages through appropriate stimulation [77]. Lin et al. discovered that the *Cordyceps militaris* polysaccharide fraction (50−250 µg/mL) reduced apoptosis in THP-1 monocytes induced by aflatoxin while promoting proliferation. Furthermore, it enhanced the expression of immunoregulatory cytokines, including IL-1β, TNF-α, IFN-γ, and IL-6 [78]. The polysaccharide SD-PK5, a linear β-(1→3)-D-glucan, demonstrated the ability to stimulate THP-1 macrophages to express IL-1β, TNF-α, and cyclooxygenase-2 (COX-2) within the concentration range of 10 to 250 µg/mL [50]. The APS is an acidic polysaccharide with a molecular weight of 576 kDa. The monosaccharide composition includes galactose (58.3%), arabinose (27.8%), xylose (7.5%), and rhamnose (6.4%). It is mainly composed of residues such as Ara*f*-(1→, →5)-Ara*f*-(1→, →4)-Gal*p*-(1→, and →4)-GalA*p*-(1→ residues. Immunological experimental results demonstrated that APSs (1–100 μg/mL) induced alterations in the morphological characteristics of THP-1 monocytes, promoting their differentiation into macrophages. Additionally, APS stimulation resulted in increased phagocytic activity and elevated expression levels of TNF-α, IL-12 p40, IL-8, TLR-2, and TLR-4 [79]. These studies demonstrate that *Cordyceps militaris* polysaccharides are capable of inducing the differentiation of monocytes into macrophages, augmenting their phagocytic activity, and stimulating the expression of immune factors.

In addition to activating macrophages, research shows that the *Cordyceps militaris* polysaccharide can promote the immune activity of lymphocytes. The polysaccharide CMPB90-1 is a heteropolysaccharide with a molecular weight of 5.8 kDa. The monosaccharide composition includes galactose, glucose, and mannose with a molar ratio of 3.04:1.00:1.45. The backbone is composed of (1→6)-linked α-d-Glc*p* and (1→3)-linked α-d-Glc*p* residues, with branching at *O*-6, consisting of (1→4)-linked β-d-Man*p* and (1→6)-linked α-d-Glc*p* residues, respectively. Immunological experimental results revealed that CMPB90-1 (250–500 μg/mL) demonstrated the ability to stimulate the proliferation of mouse spleen lymphocytes, augment the cytotoxicity of mouse natural killer cells, and enhance the secretion of IL-2 by mouse lymphocytes. Additionally, CMPB90-1 induced an upregulation in T-cell subsets, as evidenced by an increased CD4+/CD8+ T lymphocyte ratio [43]. Wu et al. isolated two acidic polysaccharides, namely, CM-jd(Y)-CPS2 and CM-jd-CPS2, from *Cordyceps militaris*. Both CM-jd(Y)-CPS2 and CM-jd-CPS2 exhibited a proliferative effect on mouse splenocytes. Moreover, they synergistically enhanced the maturation of mouse T lymphocytes and B lymphocytes induced by concanavalin A and lipopolysaccharides. Notably, CM-jd(Y)-CPS2 demonstrated a more robust stimulatory activity compared to CM-jd-CPS2. The authors inferred that this discrepancy arose from the distinctive structural features of CM-jd-CPS2, characterized by sulfation and the presence of an acetylamino group, in contrast to CM-jd(Y)-CPS2, which is a carboxylated polysaccharide. Consequently, CM-jd(Y)-CPS2 exhibited enhanced capability in establishing associations with target biomolecules through electronic interactions [49]. This indicates that the structure of the *Cordyceps* polysaccharide influences its immune activity.

### 3.2. Immunomodulatory Effects of Intracellular Polysaccharides in Animal Models

The latest research indicates that *Cordyceps militaris* polysaccharides demonstrate immunomodulatory effects across diverse animal models. These effects encompass ameliorating immune suppression induced by cyclophosphamide, restraining allergic reactions, bolstering the body’s antiviral immunity, and counteracting the immunosuppressive tumor microenvironment. 

Zhu et al. observed that a polysaccharide-rich extract from *Cordyceps militaris* (50–200 mg/kg) demonstrated the ability to enhance the spleen and thymus indices, boost splenic lymphocyte activity, strengthen macrophage function, and induce IL-2, IL-4, and IFN-γ production in cyclophosphamide-induced mice [80,81]. Wang et al. additionally demonstrated that the *Cordyceps militaris* polysaccharide fraction (17.5–70 mg/kg) could counteract cyclophosphamide-induced immunosuppression in mice. This was manifested by an increase in both spleen and thymus indices, along with the augmentation of spleen lymphocyte activity and macrophage function [82]. 

Ohta et al. reported that APSs (2–8 mg/mL) demonstrated the capacity to elevate levels of TNF-α and IFN-γ in mice infected with the influenza A virus. Additionally, APSs were observed to diminish virus titers in bronchoalveolar lavage fluid and lungs of infected mice, leading to an enhancement in mouse survival rates [39]. The *Cordyceps militaris* polysaccharide fraction (2–8 mg/mL) also demonstrated the capability to stimulate lymphocyte proliferation, boost serum antibody titers, and elevate concentrations of serum IFN-γ and IL-4 in chickens vaccinated with the Newcastle disease vaccine [83]. This observation indicates that *Cordyceps militaris* polysaccharides have the potential to enhance the immune response elicited by the Newcastle disease vaccine, suggesting their candidacy as a promising new immune adjuvant.

The polysaccharide CMPB90-1 (50–200 mg/kg) could reverse the immunosuppressive tumor microenvironment in Lewis lung carcinoma tumor-bearing mice. This reversal was achieved by reprogramming tumor-associated macrophages, shifting them from a tumor-promoting M2 phenotype to a tumor-killing M1 phenotype. Specifically, this process reversed the functional inhibition of T lymphocytes by inhibiting the programmed death-ligand 1 (PD-L1)/programmed cell death protein 1 (PD-1) axis between tumor-associated macrophages and T lymphocytes [84]. Zhong et al. reported that a selenium-rich proteoglycan extract from *Cordyceps militaris* (100–200 mg/kg) boosted immune function and hindered tumor growth by increasing lymphocyte transformation rate, enhancing macrophage clearance, and elevating the spleen index in ascites tumor-bearing mice [85].

In ovalbumin-induced asthmatic mice, the *Cordyceps militaris* polysaccharide fraction (25–100 mg/kg) suppressed the secretion of eotaxin, IL-4, IL-5, IL-13, and IFN-γ in both blood and bronchoalveolar lavage fluid. Furthermore, it decreased serum IgE levels by inhibiting the activation of the TGF-β/Smad pathway [40,86]. These findings suggest that *Cordyceps militaris* polysaccharides may hold promise as potential therapeutic agents for treating allergic asthma.

### 3.3. Immunomodulatory Effects of Extracellular Polysaccharides

Current research has identified three pure extracellular polysaccharides from *Cordyceps militaris* with immunomodulatory effects, namely, PLCM, EPS-III, and AESP-II. PLCM is a 1,6-branched glucogalactomannan with a molecular weight of 36 kDa. Results from immunological experiments demonstrated that PLCM (10–200 μg/mL) enhanced phagocytic activity and increased the secretion of NO, IL-6, and TNF-α in RAW264.7 cells by activating the MAPK and NF-κB signaling pathways [37]. EPS-III is a neutral polysaccharide with a molecular weight of 1.56 kDa. Its monosaccharide composition includes mannose, glucose, and galactose with a molar ratio of 1.68:1:1.83. The backbone consists of →4)-α-D-Gal*p*-(1→, while →3,6)-α-D-Man*p*-(1→, →4)-α-D-Man*p*-(1→, and →3)-β-D-Gal*p*-(1→ are distributed in the backbone or in the branch chains. Immunological experiment results revealed that EPS-III (225 mg/kg) could safeguard the immune organs of streptozotocin-induced diabetic mice from damage induced by high glucose, leading to an enhancement in the spleen index [44]. AESP-II is an acidic pyranose with a molecular weight of 61.52 kDa. Its monosaccharide composition includes mannose, glucuronic acid, rhamnose, galactose acid, N-acetyl-galactosamine, glucose, galactose, and arabinose with a molar ratio of 1.07:5.38:1:3.14:2.23:15:6.09:4.04. Immunological experiment results revealed that AESP-II (25–100 mg/kg) enhanced the proliferation of splenic T and B lymphocytes, raised the levels of IL-2, IL-4, and IFN-γ secreted by T lymphocytes; and increased the levels of immunoglobulins (IgG, IgM, and IgA) secreted by B lymphocytes in cyclophosphamide-induced immunocompromised mice. These effects were achieved through the activation of the p38, ERK, and JNK signaling pathways [47]. These studies demonstrate that the immunoregulatory role of *Cordyceps militaris* exopolysaccharides primarily involves enhancing the activity of macrophages and lymphocytes, as well as promoting the expression of immune factors.

## 4. The Immunomodulatory Effects of *Cordyceps cicadae* Polysaccharides

*Cordyceps cicadae* is an entomogenous fungus belonging to the *Clavicipitaceae* family and the genus *Cordyceps*. It primarily grows inside the nymphs of cicadas and has been utilized for centuries as both food and traditional medicine to address and alleviate a variety of health conditions [87]. JCH-1, JCH-2, IPS1, IPS2, and CP80-1 are five intracellular polysaccharides extracted from *Cordyceps cicadae*. Chemical structure characterization revealed that IPS1 has a molecular weight of 2400 kDa. Its monosaccharide composition comprises mannose, glucose, and galactose with a molar ratio of 1.35:6.93:1.0. The backbone consists of →4)-α-D-Glc*p* (1→ and →3,4)-α-D-Man*p* (1→ residues with a side chain consisting of T-α-D-Gal*p*. IPS2 has a molecular weight of 697 kDa, and its monosaccharide composition comprises mannose, glucose, and galactose with a molar ratio of 2.04:1.0:1.87. The backbone consists of →4)-α-D-Glc*p*-(1→, →3,4)-α-D-Man*p*-(1→, and →2,6)-α-D-Man*p*-(1→ residues, with branches also consisting of T-α-D-Gal*p* [51]. CP80-1 has a molecular weight of 25.461 kDa, and its monosaccharide composition comprises glucose, xylose, and rhamnose with a molar ratio of 19.04:8.73:1.00 [53]. The molecular weight of JCH-1 is 30.9 kDa, and its monosaccharide composition includes glucose, mannose, and galactose with a molar ratio of 1.70:1.37:1.00. JCH-2 has a molecular weight of 555.3 kDa, and its monosaccharide composition comprises glucose, mannose, and galactose with a molar ratio of 5.41:1.04:1.00 [52]. Immunological experiment results demonstrated that these polysaccharides could augment the activity of RAW264.7 macrophages, elevate phagocytosis, and induce the expression of NO and cytokines IL-6, TNF-α, and IL-1β by activating the NF-κB signaling pathway [80,81,82].

Furthermore, the exopolysaccharides of *Cordyceps cicadae* have been verified to exhibit immunomodulatory activity. Cheng et al. discovered that the *Cordyceps cicadae* exopolysaccharide fraction (12.5–100 μg/mL) could stimulate the proliferation of RAW264.7 cells and enhance NO production [88]. Kim et al. also discovered that the *Cordyceps cicadae* exopolysaccharide fraction (10–100 μg/mL) could stimulate the activity of mouse primary macrophages. This stimulation involved binding to membrane receptors such as TLR4, activating MAPK and NF-κB signaling, and increasing the production of NO, IL-1β, TNF-α, and IL-6 [89]. Kim et al. observed that the *Cordyceps cicadae* exopolysaccharide fraction (10–100 mg/mL), in addition to its immunomodulatory effects on macrophages, could promote mouse dendritic cell maturation. This enhancement was manifested by an increase in the expression of MHC-I, MHC-II, CD80, and CD86; a reduction in endocytosis; heightened cytokine production; and an increased capacity to induce allogeneic T-cell activation [85].

## 5. Immunomodulatory Effects of Polysaccharides from Other *Cordyceps* Species

### 5.1. The Cordyceps kyushuensis Polysaccharide

*Cordyceps kyushuensis* is the sole species of *Cordyceps* that grows on the larvae of *Cianis bilineata* Walker [90]. Su et al. extracted four pure polysaccharides, namely, CKPS-1, CKPS-2, CKPS-3, and CKPS-4, from *Cordyceps kyushuensis*. Their molecular weights are 7153, 5945, 5643, and 5642 kDa, respectively. The monosaccharide composition of CKPS-1 includes fructose, mannose, glucose, and galactose with a molar ratio of 1:0.92:1.09:0.72. CKPS-2, CKPS-3, and CKPS-4 consist of fructose, mannose, and galactose with molar ratios of 1:0.63:0.61, 1:1.65:1.4, and 1:2.06:1.97, respectively. Immunological experiments demonstrated that CKPS-1, CKPS-2, CKPS-3, and CKPS-4 (62.5–500 μg/mL) all induced the proliferation of mouse splenocytes, enhanced the phagocytic capacity of peritoneal macrophages, and elevated IL-2 and TNF-α expression levels [54].

### 5.2. The Cordyceps gunnii Polysaccharide

*Cordyceps gunnii* Berk., initially named after a specimen found in Tasmania, Australia, is frequently reported in China as an adulterant of the *Cordyceps sinensis* [91]. Meng et al. discovered that the *Cordyceps gunnii* polysaccharide fraction (125–500 mg/kg) could elevate the spleen and thymus indices, boost natural killer cell toxicity and lymphocyte proliferation activity, and expedite the recovery of peripheral blood white blood cells, red blood cells, hemoglobin, and platelets in cyclophosphamide-induced immunodeficient mice. Additionally, they observed an upregulation of serum IL-2, IL-12, IFN-γ, and IgG levels, along with a reduction in TGF-β levels. These effects were attributed to the activation of the TLR4/NF-κB signaling pathway [92]. Moreover, the *Cordyceps gunnii* polysaccharide fraction (50–200 mg/kg) could elevate the thymus and spleen indices, enhance macrophage phagocytosis capacity, stimulate splenocyte proliferation, and increase the expression of IFN-γ and TNF-α in H22 tumor-bearing mice. These effects contributed to the inhibition of cancer cell growth [93].

### 5.3. The Cordyceps taii Polysaccharide

*Cordyceps taii* is an entomogenous fungus native to South China [94], from which two pure polysaccharides with immunomodulatory effects, PCT-1 and CTP, are extracted. Preliminary chemical structure characterization revealed that the PCT-1 monosaccharide composition includes glucose, mannose, and galactose in a molar ratio of 5.06:4.21:1.00. In a mouse model of D-galactose-induced aging, PCT-1 (100–400 mg/kg) demonstrated the ability to enhance the proliferation of mouse T and B lymphocytes, along with an increase in IgG expression [55]. Preliminary chemical structure characterization revealed that CTP comprises glucose, galactose, and mannose with a molar ratio of 1.14:1.00:1.66 and features a series of α-(1,4) glucosidic bonds. Immunological experiment results demonstrated that the thymus index of streptozotocin-induced diabetic rats increased after treatment with CTP (400 mg/kg). It was speculated that CTP might function as an immunomodulator, potentially restoring thymus weight and enhancing the immune function of pancreatic β cells [95].

### 5.4. The Cordyceps sobolifera Polysaccharide

*Cordyceps sobolifera* parasitizes on wingless cicada nymphs, and there are currently few reports on this species [96]. The results of immunological experiments indicated that the selenium-rich extracellular polysaccharide fraction (50–200 mg/kg) could enhance the immune organ index, increase serum cytokine IL-2 and TNF-α levels, and elevate the ratio of CD8+/CD4+ T lymphocytes in colon cancer tumor-bearing mice. These effects contributed to the inhibition of tumor growth [97].

## 6. Conclusions and Perspectives

In summary, these studies demonstrate the substantial immunomodulatory effects of *Cordyceps* polysaccharides, suggesting their potential application in the treatment and amelioration of diverse diseases (Table 2). 

**Figure 1:**
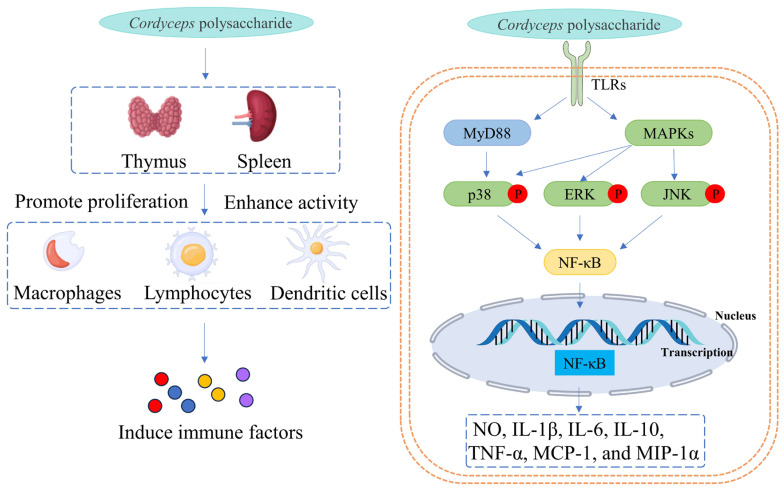
Schematic diagram of the immune regulation mechanism of Cordyceps polysaccharide.

These polysaccharides primarily originate from the widely discussed *Cordyceps sinensis* and *Cordyceps militaris*. These encompass intracellular polysaccharides extracted from fruiting bodies or mycelium, as well as extracellular polysaccharides obtained from the fermentation broth of artificially cultivated *Cordyceps*. Several studies have indicated that polysaccharides derived from *Cordyceps cicadae*, *Cordyceps kyushuensis*, *Cordyceps gunnii*, *Cordyceps taii*, and *Cordyceps sobolifera* also exhibit immunomodulatory activity. However, the number of such studies is still limited. The immunological experiment results indicate that *Cordyceps* polysaccharides have the capacity to stimulate phenotype switching and differentiation of macrophages, augment phagocytic activity, and activate the MAPK and NF-κB signaling pathways, thereby inducing the expression of various immunoactive substances such as NO, IL-1β, IL-6, IL-10, TNF-α, MCP-1, and MIP-1α (Figure 1). Furthermore, *Cordyceps* polysaccharides demonstrate the ability to induce the proliferation and activation of lymphocytes. This is achieved through the upregulation of MHC-II, CD40, CD80, and CD86 expression, as well as the induction of the expression of immunoactive substances such as IL-2, IL-12, IFN-γ, TNF-α, IL-4, IL-10, and GATA-3. *Cordyceps* polysaccharides have also been observed to promote the maturation and activation of dendritic cells, enhancing their capacity to uptake antigens and stimulate lymphocyte proliferation. Animal experiments have provided additional confirmation of the immunomodulatory effects of *Cordyceps* polysaccharides. *Cordyceps* polysaccharides have the capacity to ameliorate immune suppression induced by cyclophosphamide or radiation, reverse immune evasion prompted by tumors, and bolster the body’s antiviral capabilities.

While current research indicates immunomodulatory effects of *Cordyceps* polysaccharides, there remain certain issues that warrant further exploration. There is a limited number of studies investigating the connection between polysaccharide structure and immunomodulatory activity, despite the comprehensive exploration of the immunomodulatory effects of *Cordyceps* polysaccharides in current research. Given the close correlation between polysaccharide structure and its activity, it is of paramount significance to delve deeper into understanding the relationship between polysaccharide structure and immune activity. Secondly, current research predominantly focuses on investigating the immunomodulatory activity of *Cordyceps* polysaccharides, specifically their effect on macrophages and lymphocytes. However, there is a paucity of studies concerning other vital immune cells, such as dendritic cells and natural killer cells. There is also a need for in-depth exploration of additional mechanisms involved in immune regulation. Thirdly, the current research predominantly emphasizes cell experiments and animal studies. There is consequently a demand for additional clinical investigations to confirm the immunomodulatory effects of *Cordyceps* polysaccharides.

## Figures and Tables

**Table 1:** The pure polysaccharides with immunomodulatory effects extracted from *Cordyceps*.

Source	Name	M.W. (kDa)	Monosaccharide Composition	Structural Characteristics	Ref.
*Cordyceps sinensis*	CCP	433.788	Glc	Composed of non-reducing terminal D-glc*p*, (1→4)-linked D-g glc*p*, and (1→4,6)-linked D-glc*p* residues	[22]
	PHP	58.14	Man (2.49%), Glc (57.1%), Gal (1.43%), and GalA (0.321%)	ND	[23]
	CM-S	134.631	Gal, Glc, and Xyl with a molar ratio of 3:2:1	Main chain is (1→6)-α-d-gal, which is connected at the C2 site with (1,4→6)-α-d-glc at the C1 site	[12]
	CP2-S	1.328	Mainly composed of Glc	ND	[24]
	OSP	27.972	Xyl, Man, Glc, and Gal with a molar ratio of 2.9:6.6:166:2.6	ND	[25]
	Cordysinan	22.37	Man, Gal, and Glc with a molar ratio of 4.4:3.8:1.0	Backbone composed of 1,2-Man*p* residues	[26]
	UM01-S4	22.569	Man, Glc, Gal, and GalA with a molar ratio of 9.6:4.0:4.4:1.0	Backbone composed of α-(1→2)-Man*p* and with side chains consisting of β-(1→4)-Glc*p*, β-(1→2)-Gal*f*, terminal α-Gal*p*A and α-Man*p*	[27]
	CP-PS	12	Man, Rha, Ara, Xyl, Glc, and Gal with a molar ratio of 38.37:2.51:2.21:5.22:27.44:24.25	ND	[28]
	Cs-HK1	36	Glc and GalA with a molar ratio of 8:1	Linear backbone of (1→3)-linked α-D-Glc*p*	[29]
	EPS	104	Glc and Gal with a molar ratio of 23:1:2.6	ND	[30]
	EPS	ND	Glc and Man with a molar ratio of 99:1	Linear backbone of (1→3)-β-D-Glc*p* residues	[31]
	HSWP-2a	870.70	Glc	α-(1→4)-D-glucan	[32]
	HS002-II	44	Man, Rib, Rha, GlcA, GalA, Glc, Gal, Xyl, and Ara with a molar ratio of 6.47:2.27:1.25:0.69:0.42:65.89:16.17:2.13:4.26	Long backbone of (1→3)-linked α-d-Rib*f* units (1→4)-linked α-d-Xyl*p* units and (1→4)-linked β-d-Glc*p* units	[33]
	NCSP-50	976	Glc	Backbone of (1→4)-linked-α-D-Glc*p* with a single α-D-Glc*p* branch substituted at C-6	[34]
	PS	83	Glc, Man, Ara, and Gal with a molar ratio of 8:90:1:1	ND	[35]
	UST 2000	82	Glc, Man, and Gal with a molar ratio of 2.4:2:1	ND	[36]
	AEPS-1	36	Glc	Linear backbone of (1→3)-linked α-D-Glc*p* residues with two branches, α-D-Glc*p* and α-D-Glc*p*	[29]
*Cordyceps militaris*	CM-S	134.631	Gal, Glc, and Xyl with a molar ratio of 3:2:1	Main chain is (1→6)-α-d-Gal, which is connected at the C2 site with (1,4→6)-α-d-Glc at the C1 site	[12]
	PLCM	36	Glc	a 1,6-branched-Glc	[37]
	HCMP	6180	Ara, Gal, Glc, Man, and Xyl, with a molar ratio of 2.00:8.01:72.54:15.98:1.02	ND	[38]
	APS	576	Gal (58.3%), Ara (27.8%), Xyl (7.5%), and Rha (6.4%)	Composed of Ara*f*-(1→, →5)-Ara*f*-(1→, →4)-Gal*p*-(1→ and →4)-GalA*p*-(1→ residues	[39]
	CMP	15.94	Glc, Man, Gal, Xyl, Ara, and GlcA with a molar ratio of 81.25:21.96:13.88:3.92:3.58:1.00	ND	[40]
	CMP-W1	366	Man, Glc, and Gal with a molar ratio of 2.84:1:1.29	Main chain composed of 1→3,1→2,3,1→2,4,1→3,4,1→3,6, or 1→2,3,4 glycosidic bond	[41]
	CMP-S1	460	Man, Glc, and Gal with a molar ratio of 2.05:1:1.09	Lots of residues are 1→,1→6,1→2,1→2,6,1→4, and 1→4,6 linked	[41]
	CMP Fr II	126	Glc, Gal, and Man with a molar ratio of 56.4:26.4:17.2	Composed of the (1→4) or (1→2)-linked Glc*p* or Gal*p* residue with a (1→2) or (1→6)-linked Man*p*, Glc*p*, or Gal*p* residue as a side chain	[42]
	CMPB90-1	5.8	Gal, Glc, and Man with a molar ratio of 3.04:1.00:1.45	Backbone of (1→6)-linked α-d-Glc*p* and (1→3)-linked α-d-Glc*p* residues	[43]
	EPS-III	1.56	Man, Glc, and Gal with a molar ratio of 1.68:1:1.83	Backbone of →4)-α-D-Galp-(1→	[44]
	CMP-III	49.76	Glc, Man, and Gal with a molar ratio of 8.09:1.00:0.25	Main linkage types consist of 1→4)-α-D-Glc, 1→4,6)-α-D-Man, 1→)-α-D-Man and 1 → 2,6)-α-D-Gal	[45]
	SDQCP-1	19.3	Man, Glc, and Gal with a molar ratio of 13.3:1.0:9.7	Backbone composed of (1→2)-α-D-Man*p* and (1→ 4)-β-D-Glc*p* residues	[46]
	AESP-II	61.52	Man, GlcA, Rha, GalA, Glc, Gal, and Ara with a molar ratio of 1.07:5.38:1:3.14:15:6.09: and 4.04	ND	[47]
	CPS-1	23	Rha, Xyl, Man, Glc, and Gla in with a molar ratio of 1:6.43:25.6:16.0:13.8	(1→2) linkage Man, (1→4) linkage Xyl, and (1→2) or (1→3) linkage Rha	[48]
	CM-jd-CPS2	ND	Man, Glc, and Gla with a molar ratio of 1.52:8.53:1.00	Composed of α-glycosidic linkage	[49]
	CM-jd(Y)-CPS	ND	Man, Glc, and Gla with a molar ratio of 3.11:1.00:2.12	Composed of β-glycosidic linkage	[49]
	SD-PK5	ND	Glc	A linear β-(1→3)-D-glucan	[50]
*Cordyceps cicadae*	IPS1	2400	Man, Glc, and Gla with a molar ratio of 1.35:6.93:1.0	Backbone of →4)-α-D-Glc*p* (1→ and →3,4)-α-D-Man*p* (1 → residues	[51]
	IPS2	679	Man, Glc, and Gla with a molar ratio of 2.04:1.0:1.87	Consists of →4)-α-D-Glc*p*-(1→, →3,4)-α-D-Man*p*-(1→ and →2,6)-α-D-Man*p*-(1→ residues	[51]
	JCH-1	30.9	Man, Glc, and Gla with a molar ratio of 1.70:1.37:1.00	Composed of α-type glycosidic linkage	[52]
	JCH-2	555.3	Man, Glc, and Gla with a molar ratio of 5.41:1.04:1.00	ND	[52]
	CP80-1	25.461	Glc, Xyl, and Rha with a molar ratio of 19.04:8.73:1.00	Composed of α- and β-glycosidic linkages	[53]
*Cordyceps kyushuensis*	CKPS-1	7153	Fuc, Man, Glc, and Gal with a molar ratio of 1:0.92:1.09:0.72	Composed of α- and β-glycosidic linkages	[54]
	CKPS-2	5945	Fuc, Man, and Gal with a molar ratio of 1:0.63:0.61	Composed of α- and β-glycosidic linkages	[54]
	CKPS-3	5643	Fuc, Man, and Gal with a molar ratio of 1:1.65:1.4	Composed of α- and β-glycosidic linkages	[54]
	CKPS-4	5642	Fuc, Man, and Gal with a molar ratio of 1:2.06:1.97	Composed of α- and β-glycosidic linkages	[54]
*Cordyceps taii*	PCT-1	ND	Glc, Man, and Gal with a molar ratio of 5.06:4.21:1.00	Composed of α- and β-glycosidic linkages	[55]
	PCT	ND	Glc, Gal, and Man with a molar ratio of 1.14:1.00:1.66	Composed of series α-(1, 4) glucosidic bond	[56]

Abbreviations: Ara, arabinose; Fuc, fucose; GluA, glucuronic acid; Xyl, xylose; Man, mannose; Glc, glucose; Gal, galactose; Rha, rhamnose; GalA, galacturonic acid; GlcA, glucuronic acid. ND, not detected.

**Table 2:** The pharmacological effect of *Cordyceps* polysaccharides.

Species Name	Polysaccharide Name/Fraction	Details of Pharmacological Activity	Cell Line/Model	Dosage	Ref.
*Cordyceps sinensis*	Fraction	Activates NF-κB pathway; induces M1 phenotype; and upregulates TNF-α, IL-12, and iNOS expression while downregulating IL-10	Ana-1 cells	25–100 μg/mL	[58]
	Fraction	Activates NF-κB pathway and stimulates the release of NO and cytokines IL-1α, IL-1β, IL-10, and TNF-α	RAW264.7 cells	30–300 μg/mL	[59,60]
	Fraction	Enhances macrophage activity, induces differentiation into dendritic cells, and promotes their maturation	RAW 264.7 cells	0.1–15 μg/mL	[61]
	Fraction	Promotes proliferation and enhances the expression of CD4 and CD8	Simulated microgravity-induced murine splenic lymphocytes	25–50 μg/mL	[63]
	Fraction	Increases histone H3 acetylation, induces Foxp3 expression in regulatory T cells, and decreases IL-17 and IL-21 expression	Colons of mice induced with cyclophosphamide	200 mg/kg	[65]
	Fraction	Regulates the polarization of Th1/Th2 cells and increases IL-2, IL-12 p40, IFN-γ, TNF-α, IL-4, IL-10, GATA-3, and secretory IgA levels	Cyclophosphamide-induced mice	25–100 mg/kg	[66]
	Fraction	Stimulates IL-12, IFN-γ, IL-4, IL-13, IL-6, IL-17, IL-10, TGF-β3, TNF-α, IL-2, IL-21, T-bet, GATA-3, RORγt, and Foxp3 production	Cyclophosphamide-induced mice	25–100 mg/kg	[67]
	Fraction	Enhances lymphocyte proliferation and macrophage phagocytosis activity, reduces IL-4 and IL-17 expression, and increases IL-5 expression	Mice exposed to ^60^Co	50–200 mg/kg	[28]
	Fraction	Accelerates the recovery of white blood cells, increases the organ index of the thymus and spleen	X-ray irradiation-injured mice	100–400 mg/kg	[69]
	Fraction	Elevates thymus index, spleen index, and the number of CD4+ and CD8+ T lymphocytes and macrophages	H22 tumor-bearing mice	100–400 mg/kg	[22]
	PS	Enhances the serum levels of IgG, IgG1, and IgG2b	OVA-immunized mice	100–400 μg/mouse	[35]
	AEPS-1	Induces the production of TNF-α, IL-6, and IL-1β	RAW264.7 cells	25–250 μg/mL	[29]
	OSP	Activates MAPK and PI3K/Akt pathways and induces the production of TNF-α, IL-6, and IL-1β	RAW264.7 cells	6.25–50 μg/mL	[25]
	HS002-II	Activates NF-κB pathways and induces the production of NO, TNF-α, IL-6, and IL-1β	RAW264.7 cells	0.2785–4.4 μM	[33]
	EPS	Increases IL-12, TNF-α, and iNOS expression and upregulates capacity for antigen uptake and activation of T lymphocyte proliferation	Mouse dendritic cells	25–100 μg/mL	[72,73]
	EPS	Increases thymus and spleen indices and stimulates the release of TNF-α and INF-γ	Cyclophosphamide-induced mice	20 mg/kg	[31]
	UST 2000	Activates ERK signaling pathway and promotes proliferation and IL-2, IL-6, and IL-8 secretion	Human T lymphocytes	6.25–100 μg/mL	[36]
	PHP	Reduces the number of Th17 cells and increases the number of Treg cells	Dextran sulfate sodium-induced mice	400 mg/kg	[23]
	NCSP-50	Induces proliferation and augment the production of NO, IL-1β and TNF-α	RAW 264.7 cells	25–200 μg/mL	[34]
	NCSP-50	Increases the number of CD4+ T cells; modulates TLR expression; and increases IL-17, IL-21, and TGF-β3 levels	Cyclophosphamide-induced mice	25–100 mg/kg	[68]
	CCP	Activates TLR4/MyD88/p38 pathway and induces NO, IL-6, and TNF-α production	RAW 264.7 cells	1–400 μg/mL	[62]
	HSWP-2a	Activates p38, JNK, and NF-κB pathways and augments phagocytic activity and the production of NO, IL-1β, IL-6, and TNF-α	RAW264.7 cells	25–200 μg/mL	[32]
	UM01-S4	Activates MAPK and NF-κB pathways; boosts proliferation and phagocytic activity; and stimulates NO, IL-1β, IL-6, IL-12, and TNF-α expression	RAW 264.7 cells	0.1–3 μg/mL	[27]
	Cordysinan	Induces the release of IL-1β, IL-6, IL-10, TNF-α, MCP-1, MIP-1α, IP-10, and KC	RAW 264.7 cells	10–100 μg/mL	[26]
*Cordyceps militaris*	CP2-S	Stimulates NO production, phagocytosis, respiratory burst activity, and the secretion of IL-1β and IL-2	RAW264.7 cells	50–500 μg/mL	[24]
	Fraction	Promotes proliferation and enhances the expression of IL-1β, TNF-α, IFN-γ, and IL-6	THP-1 and EL-4 T cells induced by aflatoxin	50−250 µg/mL	[78]
	Fraction	Increases spleen and thymus indices and spleen lymphocyte activity and macrophage function	Cyclophosphamide-induced mice	17.5–70 mg/kg	[82]
	Fraction	Stimulates lymphocyte proliferation, boosts serum antibody titers, and elevates serum IFN-γ and IL-4 levels	Chickens vaccinated with the Newcastle disease vaccine	2–8 mg/mL	[83]
	Fraction	Suppresses the secretion of eotaxin, IL-4, IL-5, IL-13, IFN-γ, and IgE	Ovalbumin-induced asthma mice	25–100 mg/kg	[40,85]
	Fraction	Stimulates proliferation and enhances NO production	RAW264.7 cells	12.5–100 μg/mL	[88]
	Fraction	Activates MAPK and NF-κB signaling and increases the production of NO, IL-1β, TNF-α, and IL-6	Mouse primary macrophages	10–100 μg/mL	[89]
	Fraction	Increases MHC-I, MHC-II, CD80, and CD86 expression and induces allogeneic T-cell activation	Mouse dendritic cells	10–100 mg/mL	[98]
	Polysaccharide-rich extract	Boosts splenic lymphocyte activity; strengthens macrophage function; and induces IL-2, IL-4, and IFN-γ expression	Cyclophosphamide-induced mice	50–200 mg/kg	[80,81]
	Selenium-rich proteoglycan extract	Increases lymphocyte transformation rate, enhances macrophage clearance, and elevates spleen coefficient	Ascites tumor-bearing mice	100–200 mg/kg	[85]
	CMP-Fr-II	Increases NO, TNF-α, and IL-1β expression	RAW264.7 cells	1000 µg/mL	[42]
	CMP-III	Activates MAPK and NF-κB pathways and induces NO, TNF-α, and IL-6 production	RAW264.7 cells	50–500 μg/mL	[45]
	SDQCP-1	Induces M1 polarization and stimulates NO, TNF-α, IL-6, and IL-10 release	RAW264.7 cells	50−250 µg/mL	[46]
	SD-PK5	Stimulates IL-1β, TNF-α, and COX-2 expression	THP-1 cells	10−250 µg/mL	[50]
	APS	Promotes differentiation into macrophages and increases phagocytic activity and TNF-α, IL12 p40, IL-8, TLR-2, and TLR-4 expression	THP-1 cells	1–100 μg/mL	[79]
	APS	Elevates TNF-α and IFN-γ expression and diminishes virus titers in bronchoalveolar lavage fluid and lungs	Mice infected with influenza A virus	2–8 mg/ml	[39]
	CMPB90-1	Stimulates proliferation and enhances the secretion of IL-2	Mouse spleen lymphocytes	250–500 μg/mL	[43]
	CMPB90-1	Shifts tumor-promoting M2 phenotype macrophages to a tumor-killing M1 phenotype	Lewis lung carcinoma tumor-bearing mice	50–200 mg/kg	[51]
	PLCM	Activates MAPK and NF-κB pathways; enhances phagocytic activity; and increases NO, IL-6, and TNF-α expression	RAW264.7 cells	10–200 μg/mL	[37]
	EPS-III	Safeguards the immune organs of from damage induced by high glucose, leading to an enhancement in spleen index	Streptozotocin-induced diabetic mice	225 mg/kg	[44]
	AESP-II	Enhances T and B cell proliferation and raises IL-2, IL-4, IFN-γ, IgG, IgM, and IgA levels	Cyclophosphamide-induced mice	25–100 mg/kg	[47]
	JCH-1 and JCH-2	Elevate phagocytosis and induce NO, IL-6, TNF-α, and IL-1β expression	RAW264.7 cells	5–25 μg/mL	[52]
	IPS1 and IPS2	Elevate phagocytosis and induce NO, IL-6, TNF-α, and IL-1β expression	RAW264.7 cells	50–400 μg/mL	[51]
	CP80-1	Elevates phagocytosis and induces NO, IL-6, TNF-α, and IL-1β expression	RAW264.7 cells	25–100 μg/mL	[53]
*Cordyceps gunnii*	Fraction	Elevates the spleen and thymus index, boosts natural killer cell toxicity and lymphocyte proliferation activity, and expedites the recovery of peripheral blood cells	Cyclophosphamide-induced mice	125–500 mg/kg	[92]
	Fraction	Elevates thymus and spleen indices, enhances macrophage phagocytosis capacity, and stimulates splenocyte proliferation	H22 tumor-bearing mice	50–200 mg/kg	[93]
*Cordyceps taii*	PCT-1	Enhances proliferation and increases IgG expression	Mouse T and B lymphocytes	100–400 mg/kg	[55]
	CTP	Increases thymus index	Streptozotocin-induced diabetic rats	400 mg/kg	[95]
*Cordyceps sobolifera*	Fraction	Enhances immune organ index, increases IL-2 and TNF-α levels, and elevates ratio of CD8+/CD4+ T cells	Colon cancer tumor-bearing mice	50–200 mg/kg	[97]
*Cordyceps kyushuensis*	CKPS-1, CKPS-2, CKPS-3, and CKPS-4	Induce proliferation, enhance phagocytic capacity, and elevate the levels of IL-2 and TNF-α in serum	Mouse splenocytes and peritoneal macrophages	62.5–500 μg/mL	[54]

## Data Availability

Not applicable.
